# Oral micronized progesterone for perimenopausal night sweats and hot flushes a Phase III Canada-wide randomized placebo-controlled 4 month trial

**DOI:** 10.1038/s41598-023-35826-w

**Published:** 2023-06-05

**Authors:** Jerilynn C. Prior, Andrea Cameron, Michelle Fung, Christine L. Hitchcock, Patricia Janssen, Terry Lee, Joel Singer

**Affiliations:** 1 Centre for Menstrual Cycle and Ovulation Research, Endocrinology, University of British Columbia (UBC), 2775 Laurel Street, Suite 4111, Vancouver, BC Canada; 2 School of Population and Public Health, UBC, Vancouver, Canada; 3 Etobicoke General Hospital, William Osler Health System, Etobicoke, ON Canada; 4 British Columbia Women’s Health Research Institute, Vancouver, Canada; 5 Hitchcock Consulting, Oakville, ON Canada; 6 Centre for Health Evaluation and Outcome Sciences, Vancouver, BC Canada; 7 Endocrinology and Metabolism, Department of Medicine, UBC, Vancouver, BC Canada

**Keywords:** Endocrinology, Clinical trial design, Reproductive signs and symptoms

## Abstract

This study tested progesterone for perimenopausal hot flush ± night sweat (vasomotor symptom, VMS) treatment. It was a double-blind, randomized trial of 300 mg oral micronized progesterone@bedtime versus placebo for 3-months (m) after a 1-m untreated baseline during 2012/1–2017/4. We randomized untreated, non-depressed, screen- and baseline-eligible by VMS, perimenopausal women (with flow within 1-year), ages 35–58 (n = 189). Participants aged 50 (± SD = 4.6) were mostly White, educated, minimally overweight with 63% in late perimenopause; 93% participated remotely. The 1° outcome was 3rd-m VMS Score difference. Participants recorded VMS number and intensity (0–4 scale)/24 h on a VMS Calendar. Randomization required VMS (intensity 2–4/4) of sufficient frequency and/or ≥ 2/week night sweat awakenings. Baseline total VMS Score (SD) was 12.2 (11.3) without assignment difference. Third-m VMS Score did not differ by therapy (Rate Difference − 1.51). However, the 95% CI [− 3.97, 0.95] *P* = 0.222, did not exclude 3, a minimal clinically important difference. Women perceived progesterone caused decreased night sweats (*P* = 0.023) and improved sleep quality (P = 0.005); it decreased perimenopause-related life interference (*P* = 0.017) without increased depression. No serious adverse events occurred. Perimenopausal night sweats ± hot flushes are variable; this RCT was underpowered but could not exclude a minimal clinically important VMS benefit. Perceived night sweats and sleep quality significantly improved.

## Introduction

In population-based data, perimenopausal women, who have menstruated within 1-year, are as likely to experience night sweats and/or hot flushes (vasomotor symptoms, VMS) as postmenopausal women^1^ and often have more severe VMS^2^. However, perimenopausal women also commonly experience heavy menstrual bleeding^3^, sore breasts^4^ and mood swings^5^, that, along with VMS, have not been shown to be effectively and safely treated in randomized placebo-controlled trials (RCT) of Menopause Hormone Therapy (MHT), which is universally recommended by guidelines^6–8^. Although Fezolinetant is a very promising, effective and safe VMS therapy, it has only to our knowledge, been studied in animals and *postmenopausal* women^9,10^. However, kisspeptin/neurokinin B/dynorphin-interventions have complex and incompletely understood hypothalamic reproductive actions^11,^ thus, it is necessary to test Fezolinetant for VMS effectiveness and safety in perimenopausal women before it will be proven relevant to treatment of perimenopausal VMS.

VMS in *postmenopausal* women are effectively treated by MHT based on a meta-analysis of RCTs^12^. In the published literature we have found that only 16 women in late perimenopause have been investigated for VMS during MHT treatment^13^. The results of perimenopausal women were not reported separately from the 16 in early postmenopause in the 1-year Herbal Alternatives for Menopause Trial that randomized 32 women to MHT^13^. MHT was significantly more effective for VMS than placebo^13^. However, a quarter discontinued MHT due to adverse effects (“menstrual disturbances” in 59%; breast pain in 16%) or lack of effectiveness^13^. These data seem insufficient to support a universal policy to prescribe MHT for *perimenopausal* VMS.

We have only found two RCT that have tested estrogen-based VMS treatments solely in perimenopausal women. The first (1997), was a 6-cycle industry-sponsored RCT of a 20-µg ethinyl estradiol oral combined hormonal contraceptive (CHC) with 132 enrolled perimenopausal women of whom 74 experienced hot flushes^14^. However, documented CHC and placebo hot flash "differences were not statistically significant^14^" (page 143). The second (2015), was a pilot RCT in 38 symptomatic perimenopausal women with *regular* cycles; only 10 of whom experienced hot flushes^15^. This trial randomized women to transdermal estradiol or placebo after 90 days of wearing a levonorgestrel-releasing IUD^15^. There was no statistical difference in the prevalence of VMS on estrogen versus placebo at 50-days^15^. Neither of these perimenopausal VMS trials documented night sweats^14,15^ that appear to start early in perimenopause^4,16^ and may be more physiologically disruptive^17^. Therefore, there are currently no clear, current understandings of how to best and most safely treat the perimenopausal VMS and sleep problems for which midlife women most commonly seek treatment^18^.

Progesterone and estradiol complement or counterbalance each other’s effects in most tissues and cells^19,20^. In the brain, progesterone decreased central inflammation and oxidative stress in animal data^21^; progesterone in a human RCT study lessened anxiety^22^ and improved sleep^23,24^. Both estradiol and progesterone appear to act on VMS, at least in part, by restoring the narrowed thermoneutral zone toward normal^25^.

Based on the following evidence, we hypothesized that oral micronized progesterone would be an effective, well tolerated treatment for perimenopausal VMS^26^:

A) Progesterone was superior to placebo in *postmenopausal* community-dwelling women (n = 132) in a 3-month VMS RCT, without serious adverse events^27^ nor negative effects on cardiovascular biomarkers^28^.
B) The 46 postmenopausal women in this RCT who had Severe VMS (> 50 moderate-severe VMS/week)^29^ showed a greater VMS reduction than the whole cohort^30^.
C) Abrupt discontinuation of progesterone produced no rapid increase in VMS over 1-month in postmenopausal women^30^, whereas stopping estrogen-based VMS MHT caused a quarter of women to experience clinically problematic increases in, or “rebound” VMS^31^.
D) Progesterone levels are importantly lower in perimenopausal than in premenopausal women^32^.

Thus, progesterone VMS treatment could be considered a “replacement-type” therapy in still-menstruating perimenopausal women^26^.

We tested the hypothesis that progesterone would be effective for perimenopausal VMS with an RCT in community dwelling women who had menstruated within 1-year and who reported bothersome night sweats and/or hot flushes/flashes.

## Methods

### Trial design

This study was a double blind, Phase III, single centre (University of British Columbia, Vancouver, BC) trial with Canada-wide recruitment, of a randomized, placebo-controlled parallel design with 1:1 experimental allocation and 1-month (m) untreated baseline followed by 3-m on experimental therapy. The trial tested oral micronized progesterone (3 × 100 mg spherical capsules at bedtime daily) versus identical placebo. This RCT was active from January 2012 to April 2017. Results were podium-presented at an international meeting^33^.

### Clinical ethics board approval

The University of British Columbia Clinical Research Ethics Board approved the original protocol (*H10-02975*) and its subsequent major amendment from local to national (remote participation) recruitment. The amended protocol was also ethics approved after, with assistance from the Data Safety and Monitoring Committee (DSMC), we obtained Health Canada permission to remove mandatory mammogram and clinical breast examination screening within 1-year prior to enrolment, and to truncate to the 8-item Daily Perimenopause Hot Flush and Night Sweat Calendar© (Supplemental Figure 1) from the 23-item Daily Perimenopause Diary©^4^ for primary outcome documentation. All participants provided written, informed consent.

**Figure 1:**
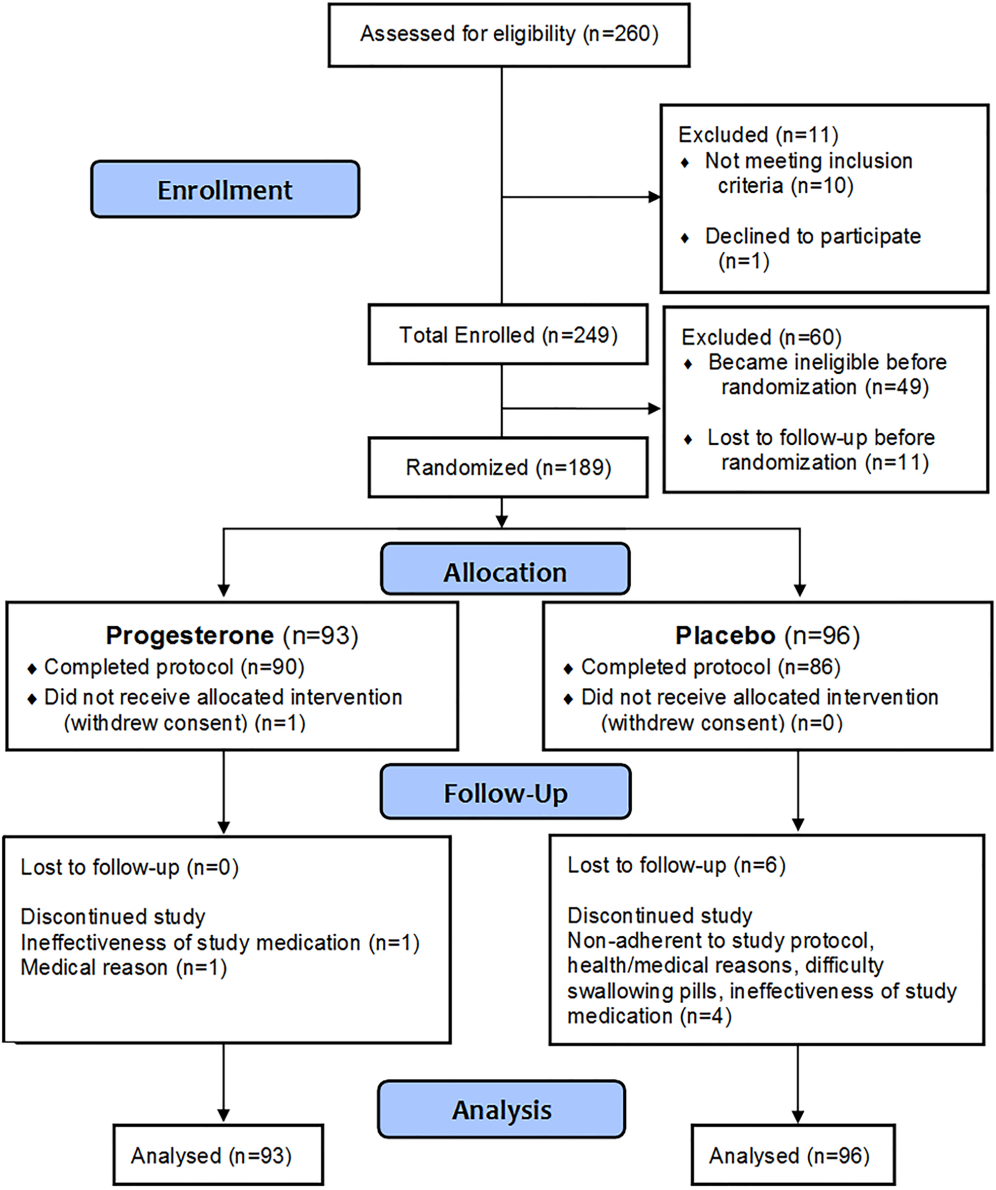
Consort figure indicating the flow of participants through the randomized controlled trial of oral micronized progesterone for perimenopausal hot flushes and night sweats (Vasomotor Symptoms).

### The protocol

Besides registration (ClinicalTrials.gov #NCT01464697, 31/10/2011), the protocol and its amendments are accessible here: https://dx.doi.org/10.14288/1.0363242. A Data Safety and Monitoring Committee (DSMC) met semi-annually to provide trial oversight. This study was conducted according to all applicable and Good Clinical Practice Guidelines and the Declaration of Helsinki.

### Participants

We recruited participants from the community through poster advertisements at community centers, coffee shops, medical, mammographic and naturopathic clinics; through ads in national magazines and on women’s health webpages (www.cemcor.ubc.ca) and through email and social media. Enrolment was complete in April, 2017.

Eligible women were aged 35–58 years, could read and write English, had experienced at least one menstruation in the previous year, were willing to document daily VMS and had problematic VMS by screening questionnaire. During a 1-m untreated baseline they were required to have an average of 4 moderate-to-severe VMS per 24-h day for at least two out of four weeks (given perimenopausal VMS cyclicity^4^) or at least 56 VMS per four-weeks (criteria similar to a previous RCT^13^) to be randomized. Based on clinical experience and documented negative night sweat physiology^17^, women were also eligible if reporting that night sweats had caused them to awaken twice weekly or more often. Each woman with breast cancer, or having a first-degree relative with it required a normal mammogram and clinical breast examination within 12 months of recruitment.

Exclusion criteria included being postmenopausal, having had a hysterectomy and/or bilateral ovariectomy, peanut allergy (peanut oil, at that time but not currently, was an experimental drug excipient), planned pregnancy/fertility treatment, breastfeeding, or MHT or CHC use within six months. A few women (< 10) wearing a progestin-releasing IUD or treated with low dose (≤ 20 mg/d) transdermal progesterone were enrolled if meeting VMS enrolment criteria and they agreed to continue consistent IUD/transdermal progesterone use throughout the RCT.

At the request of the DSMC, we screened women for depression by the Personal Health Questionnaire (PHQ-9)^34^ due to its association with VMS^35^; we excluded women with PHQ-9 ≥ 15 if, after clinical assessment, they required follow-up and/or depression therapy. We committed to provide participants with their own results, and to reveal their individual treatment assignments soon after they completed the trial (while preserving investigator and participants’ blinding). We have shared study results with participants through a password protected section of the CeMCOR website.

### Randomization and masking

Randomization was stratified by ‘Early’ Perimenopause (EP) defined as regular^15,36,37^ or irregular menstrual cycles^38^, and ‘Late’ Perimenopause (LP) identified as those having a cycle length of ≥ 60 days, or a skipped cycle within the last year^38^.

We allocated consenting participants to treatment (oral micronized progesterone 300 mg at bedtime or identical placebo) via permuted block randomization generated using Statistical Analysis Software® by a statistician (JS) unassociated with trial conduct. The trial pharmacist allocated participants through a password-protected randomization website.

### Procedures and protocol changes

Initial on-site Vancouver recruitment was slow. Some women reported not wanting the hassle of appointments for clinical breast exams and mammograms. Therefore, we revised the protocol and obtained ethical and DSMC approval for remote participation which facilitated trans-Canadian recruitment. We obtained approval from Health Canada to remove the requirement for breast cancer screening. Safety and effectiveness measures included telephone/video conference screening interview and full baseline questionnaire; each remote participant personally signed for the couriered experimental medication. Study communications used email, secure fax, telephone or web-conferencing. Participants returned unused medications in a provided stamped return-addressed, padded postal envelope.

With study enquiry, the coordinator spoke with the potential participant and provided her with the consent form. When each gave *verbal* consent, the coordinator undertook telephone-screening. Women with qualifying VMS who remained interested, provided *written* consent and began the 1-m baseline assessment (‘run-in.’).

Participants initially recorded VMS in the Daily Perimenopause Diary^4^. On DSMC instruction, this was truncated and re-named the Daily Perimenopause Hot Flush and Night Sweat Calendar© (Supplemental Figure 1). Using an online video, https://cemcor.ca/PuzzleofPerimenopause, plus personal instruction prior to starting the run-in, we taught participants to record the number and intensity of VMS when awake (hot flushes) and sleeping (night sweats). They recorded VMS *intensity* using a 0–4 scale; a score of 1 was a night sweat which did not awaken them or a perceived hot flush that required no action; VMS intensity scores of 2–4 were considered moderate-severe, involved increasing degrees of sweating, fanning/clothing removal plus awakening if they occurred during sleep.

We re-assessed trial eligibility using baseline-m VMS experiences. We randomized those remaining eligible (as described above) to experimental therapy for 3-m. We contacted participants monthly to assess their well-being, monitor adherence and systematically make open-ended inquiries about any adverse events. On the final day of experimental therapy, participants completed the secondary outcome, “Women’s Perceived Change Questionnaire”^27^ by recording changes in overall night sweats and hot flushes on a − 5 to + 5 scale.

At RCT completion, participants returned completed Calendars, Women’s Perceived Change Questionnaire (inquiring about trial changes in hot flushes, night sweats, sleep quality and vaginal flow), repeated the PHQ-9, and the Perimenopausal Interference Questionnaire (see *Outcomes*) plus returned unused study capsules. Adherence was assessed by capsule counts and review of Calendar-recorded medication-use. Enrolment and study conduct continued until available funding was exhausted. As each participant completed the trial, they were individually notified by letter from the trial pharmacist of the therapy they had been taking. Each agreed not to share this information with other participants nor with the investigators.

### VMS and other outcomes

The primary outcome, VMS Score during the 3rd month by therapy assignment, was adjusted for baseline VMS Score. The daily VMS Score (previously used in many menopause VMS trials)^39^ was computed as ([# night sweats x intensity] + [# daytime hot flushes x intensity]) and summarized as daily averages for baseline and the three 1-m study periods. The secondary outcomes, obtained on the last therapy day, assessed Women’s Perceived Changes^27^ in night sweat and hot flushes, sleep quality^27^ and menstrual flow. Other secondary outcomes included assessment of Calendar-recorded sleep problems and anxiety (0–4 scale) during baseline and the 3rd month, and the Perimenopause Interference Questionnaire^40^ that used a 10 cm line (none to maximal) to indicate physical and mental/emotional perimenopausal change-interference with usual activities.

### Statistical considerations

Previous studies in menopausal women found a VMS Score of 3 points less than placebo to be a *minimal clinically important difference*^39^. Using the interpersonal assessment method of Redelmeier^41^, we have confirmed this minimal clinically important difference with perimenopausal women (unpublished Centre for Menstrual Cycle and Ovulation data). Based on a 4-cycle pilot study in 28 women in Very Early Perimenopause^42^ with night sweats but regular cycles (personal communication, CL Hitchcock), we estimated a 20% greater VMS standard deviation (SD) in perimenopausal than in postmenopausal women^27^. We determined 175 participants would be required to detect a 3-point group difference using a two-tailed alpha of 0.05 and power of 0.80, assuming a SD of 6.2 and 20% discontinuation^43^.

Because no previously powered perimenopausal VMS RCT provided guidance, we performed a pre-planned, blinded, mid-recruitment evaluation of the final VMS Score SD during the 3rd m; we adjusted this SD for its correlation with baseline VMS Score SD. This showed an adjusted SD of 8.0. In consultation with the DSMC, the completing women’s target recruitment increased to 228. We secured funds to extend recruitment for an additional year.

Statistical analysis was per Statistical Plan by T Lee under J Singer’s direction. We compared VMS Scores at 1-m intervals between experimental groups using regression analysis adjusted for Early/Late perimenopause (EP/LP) status and average daily baseline VMS Score. The data remained skewed despite transformation; based on normality assumptions, we could not employ linear regression. Due to overly dispersed data, we utilized log-linear negative binomial regression adjusted for EP/LP phases and the log of the average daily baseline VMS Score.

We assessed the treatment effect by VMS Score as the ratio of the mean Score between the two experimental groups (*rate ratio*; RR) with 95% Confidence Intervals (95%CI). To make the results easier to understand, we converted the adjusted RR into the mean rate difference (RD) using the marginal standardization technique^44^. We used the Delta method^45^ to compute the 95% CI. We similarly analyzed VMS number and intensity. The Perimenopause Interference Questionnaire^40^ and PHQ-9^29^ were analyzed using linear regression adjusted for EP/LP status and results presented as mean difference.

Primary analysis was by Intention­to­Treat (ITT). We imputed missing data points using multiple imputation (100 imputations) by a fully conditional specification method with the predictive mean matching option (5 closest observations) to avoid imputing negative values. Variables included in the imputation model were: average baseline and study treatment VMS Score, experimental group, Early (EP)/Late (LP) Perimenopause status^1^, age^46^, body mass index (BMI)^47^, current smoking^48^, education^49^ and scores from other questionnaires that could potentially be correlated with the VMS Score. These included baseline and follow-up monthly average of Calendar rating of sleep problems and anxiety, randomization and final visit Scores from the secondary outcomes: EP/LP, Perimenopause Interference Questionnaire^40^ and PHQ-9^29^. We performed a Per Protocol (PP) sensitivity analysis for participants with ≥ 7-days of Calendar data during baseline and 3rd-month (n = 176).

“Women’s Perceived Change^27^” assessed change in overall VMS (intensity and frequency) for hot flushes and night sweats, sleep quality, and menstrual flow between experimental groups on a − 5 to + 5 scale with zero indicating no change, utilizing the Wilcoxon rank-sum test. Statistical software included SAS 9.4 (SAS Institute Inc., Cary, NC) and R 3.3.3 (R Foundation for Statistical Computing, Vienna, Austria).

## Results

### Participants

We screened 260 women for eligibility of whom 249 were screen-qualified (Fig. 1); 49 women became ineligible due to an insufficient baseline-m VMS; 11 were lost to follow-up. Of 189 randomized women (93 progesterone, 96 control), 176 (93%) completed the study. Women in the experimental therapy groups were similar at baseline including in screening record of their shortest and longest cycle lengths during the past year (Table 1). Women from seven of 10 Canadian provinces and two of three territories participated, had average ages in the late 40 s to early 50 s, were just above a normal BMI range and 87% were White; two-thirds were in Late Perimenopause [LP (n = 120)] with one-third in Early Perimenopause [EP (n = 56)].

**Table 1:** Baseline and demographic variables in the progesterone for perimenopausal night sweats and hot flushes (Vasomotor Symptom, VMS) randomized controlled trial. Data are shown as mean and SD or number (%). There were no significant differences between experimental groups.

Variable	All (n = 189)	Progesterone (n = 93)	Control (n = 96)
*Age*			
Years	49.9 (4.6)	49.4 (5.0)	50.4 (4.2)
*Body mass index*			
kg/m^2^	26.7 (5.9)	27.1 (6.2)	26.3 (5.5)
*Perimenopause category, n (%)*			
Early – never skipped a period	63 (33.3)	29 (31.2)	34 (35.4)
Late – ≥ 1 > 60-day cycle	126 (66.7)	64 (68.8)	62 (64.6)
*Type of participation, n (%)*			
In-person	13 (6.9)	5 (5.4)	8 (8.3)
Remote	176 (93.1)	88 (94.6)	88 (91.7)
*Ethnicity/Race, n (%)*			
Caucasian	165 (87.3)	83 (89.2)	82 (85.4)
Chinese	6 (3.2)	1 (1.1)	5 (5.2)
Other	18 (9.5)	9 (9.7)	9 (9.4)
*Education, n (%)*			
University degree	108 (57.1)	49 (52.7)	59 (61.5)
*Employment status, n (%)*			
Employed full time	124 (65.6)	62 (66.7)	62 (64.6)
Employed part time	31 (16.4)	16 (17.2)	15 (15.6)
Unemployed	11 (5.8)	5 (5.4)	6 (6.3)
Homemaker (full time)	11 (5.8)	5 (5.4)	6 (6.3)
Retired	7 (3.7)	3 (3.2)	4 (4.2)
Other	5 (2.7)	2 (2.2)	3 (3.1)
Cigarette lifetime use (daily for at least 6 months) n (%)	74 (39.2)	43 (46.2)	31 (32.3)
Current Cigarette use, n (%)	7 (3.7)	3 (3.2)	4 (4.2)
*VMS S core–baseline month*			
Mean per night and day (SD)	12.2 (11.3)	12.1 (10.5)	12.3 (12.2)
*VMS frequency–baseline month*			
Mean per night and day (SD)	4.9 (3.8)	4.9 (3.6)	4.9 (4.0)
*VMS intensity–baseline month*			
Mean per night and day (SD)	2.3 (0.7)	2.3 (0.7)	2.3 (0.7)
> *50 VMS with intensity* ≥ *2, per week, n (%)*	35 (18.5)	19 (20.4)	16 (16.7)
*4* + *VMS/24-h day with intensity* ≥ *2 on average for at least 2/4 weeks, n (%)*	86 (45.5)	41 (44.1)	45 (46.9)
*56* + *VMS with intensity* ≥ *2 over 4 weeks*			
n (%)	127 (67.2)	66 (71.0)	61 (63.5)
Awakened by night sweats ≥ 2x/week on average, n (%)	186 (98.4)	91 (97.8)	95 (99.0)
*Personal ealth questionnaire-9 (PHQ-9)*			
Median (interquartile range, IQR)	6.0 (3.0, 10.0)	6.0 (3.0, 10.0)	7.0 (4.0, 9.0)
*Perimenopause interference questionnaire*			
Median (IQR)	33.5 (20.5, 51.3)	35.5 (18.5, 52.5)	32.0 (22.0, 50.0)
Typical cycle length (over the adult lifetime)–days, median (IQR)	28 (28, 30)	28 (28, 30)	28 (28, 30)
Longest cycle length over the past year– days, median (IQR)	70 (40, 180)	75 (45, 180)	63 (37, 180)
Shortest cycle length over the past year – days, median (IQR)	21 (15, 28)	21 (18, 28)	21 (14, 28)

### Vasomotor Symptom Score, frequency and intensity

**Figure 2:**
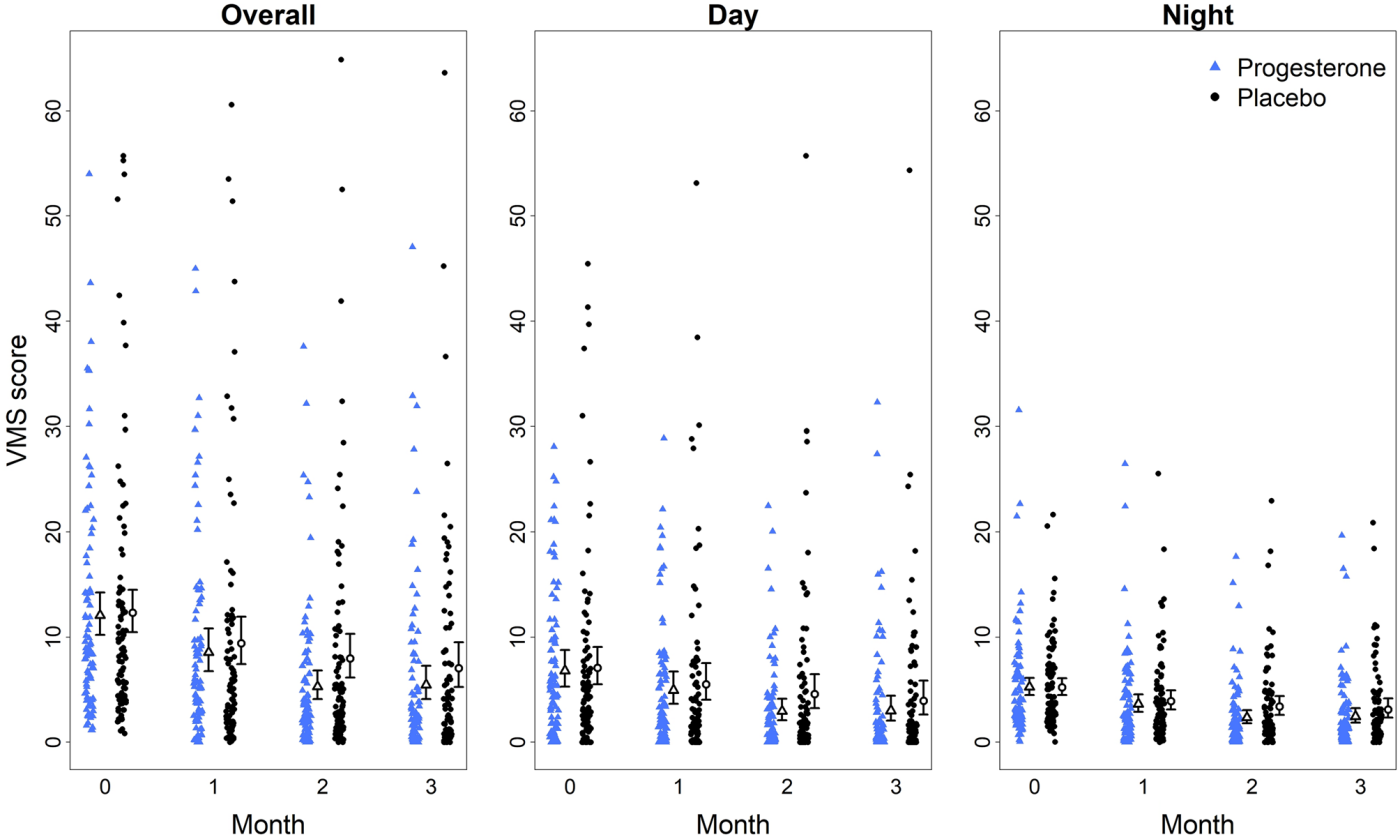
Dot-plot by experimental therapy (progesterone (n = 93)—blue triangle ∆, placebo (n = 96)—black circle $\bullet$) for Vasomotor Symptom Score* (VMS Score) and for Day and Night Sweat VMS Scores. *The VMS Score was calculated using the number times the intensity of hot flushes added to night sweats; 95% confidence interval for the mean was based on negative binomial distribution. On the X axis “0” is the 1-month baseline, with months 1 through 3 being consecutive trial periods.

The mean (SD) baseline VMS Score for all participants was 12.2 (11.3). At baseline, the overall average *frequency* of VMS/24-h day was 4.9 (3.8); average VMS *intensity* was 2.3 (0.7) on a 0–4 scale. *Daytime* VMS Scores were higher than *night sweat* Scores (Fig. 2). These data show the four months’ data as scatterplots to illustrate the variability of VMS in *peri*menopause. VMS Scores decreased over time in both experimental groups (Fig. 2).

VMS Score therapy differences were not statistically significant in the 3rd-m (RR 0.79 [95% CI 0.54, 1.15], RD [rate difference] -1.51 [95% CI − 3.97, 0.95]; *P* = 0.222) (Table 2). Also, in the 3rd-m, neither VMS frequency (RR = 0.80 [95% CI: 0.58, 1.11], *P* = 0.179) nor VMS intensity (RR = 0.89 [95% CI: 0.69, 1.15], *P* = 0.386) differed by experimental group (Table 2). However, the 95% CI of the rate difference *could not exclude a minimally important clinical VMS benefit* (*i.e.* − 3.97). VMS Score results from the *Per Protocol* sensitivity analysis in 176 women were similar: RR 0.78 [95% CI 0.53, 1.13], RD − 1.58 [95% CI − 4.00, 0.84], *P* = 0.191; VMS frequency: RR = 0.78 [95% CI: 0.57, 1.08], *P* = 0.135; VMS intensity: RR = 0.89 [95% CI: 0.68, 1.16], *P* = 0.386).

**Table 2:** Vasomotor symptom (VMS) Outcomes reported as VMS Score^#^, frequency (actual number/24-h day) and Intensity (on a 1–4 scale on which ≥ 2 involves sweating), PHQ-9 and Perimenopause Interference Questionnaire in intent to treat analysis in Perimenopausal Women Randomized to Oral Micronized Progesterone or Placebo. Data are reported as mean and standard deviation (SD). Risk ratio (RR) and risk difference (RD) are also presented.

Variables	All (n = 189)	Progesterone (n = 93)	Control (n = 96)	RR (95%CI)* RD (95%CI)*	*P
VMS Score –baseline	12.2 (11.3)	12.1 (10.5)	12.3 (12.2)	0.96 (0.77, 1.18) − 0.54 (− 3.11, 2.03)	0.680
VMS Score – 1st month	9.0 (11.0)	8.6 (9.5)	9.4 (12.3)	1.02 (0.77, 1.35) 0.15 (− 2.33, 2.64)	0.903
VMS Score – 2nd month	6.6 (9.4)	5.3 (7.0)	8.0 (11.2)	0.74 (0.53, 1.01) − 1.96 (− 4.03, 0.12)	0.060
VMS Score – 3rd month	6.2 (9.3)	5.5 (8.2)	7.1 (10.4)	0.79 (0.54, 1.15) − 1.51 (− 3.97, 0.95)	0.222
VMS frequency – baseline month per 24-h day	4.9 (3.8)	4.9 (3.6)	4.9 (4.0)	0.97 (0.81, 1.17) − 0.12 (− 1.01, 0.76)	0.783
VMS frequency –1st month per 24-h day	3.7 (3.7)	3.5 (3.2)	3.9 (4.2)	0.99 (0.79, 1.25) − 0.03 (− 0.86, 0.80)	0.951
VMS frequency – 2nd month per 24-h day	2.9 (3.2)	2.5 (2.6)	3.2 (3.7)	0.81 (0.61, 1.06) − 0.60 (− 1.35, 0.16)	0.116
VMS frequency – 3rd month per 24-h day	2.7 (3.2)	2.4 (2.7)	3.0 (3.7)	0.80 (0.58, 1.11) − 0.60 (− 1.50, 0.29)	0.179
VMS intensity – baseline	2.3 (0.7)	2.3 (0.7)	2.3 (0.7)	0.98 (0.90, 1.07) − 0.04 (− 0.24, 0.15)	0.671
VMS intensity – 1st month	1.8 (1.0)	1.8 (1.0)	1.8 (0.9)	1.06 (0.89, 1.26) 0.10 (− 0.22, 0.42)	0.539
VMS intensity –2nd month	1.6 (1.0)	1.4 (1.0)	1.7 (1.0)	0.85 (0.68, 1.06) − 0.26 (− 0.60, 0.08)	0.138
VMS intensity – 3rd month	1.4 (1.0)	1.4 (1.0)	1.5 (1.0)	0.89 (0.69, 1.15) − 0.17 (− 0.56, 0.22)	0.386
Perimenopause Interference Questionnaire	25.3 (20.2)	22.6 (18.3)	28.2 (21.7)	− 6.65 (− 12.11, − 1.18)^	**0.017**
Personal Health Questionnaire 9 (PHQ-9)	5.1 (4.4)	4.8 (4.4)	5.4 (4.3)	− 0.40 (− 1.57, 0.77)^	0.501

^#^VMS Score is the daytime number X intensity plus the nighttime number X intensity.

*Based on negative binomial regression adjusted for perimenopause phase (EP/LP) and log of the average daily run-in VMS Score (when applicable). Please refer to *Statistical Considerations*.

^Based on linear regression adjusted for perimenopausal phase status (EP/LP). Results presented as mean difference. Bolded *P* values are statistically significant

### Women’s perceived VMS changes

**Figure 3:**
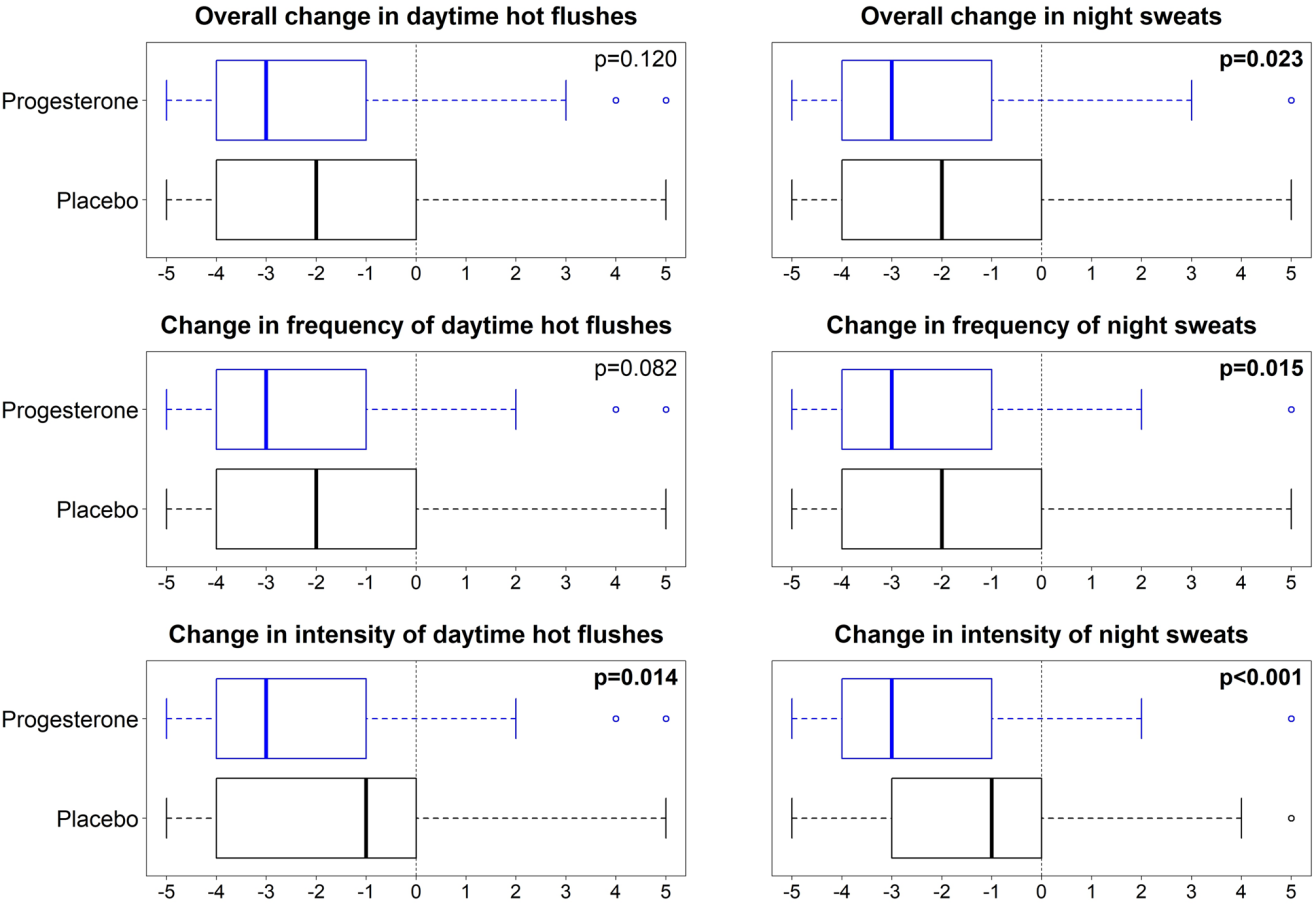
Perimenopausal Women’s Perceived Change in Day and Night Vasomotor Symptoms by Progesterone (blue) or Placebo (black) 3-Month Randomized Therapy. The solid line is the median with the box including 50% (25th to 75th percentiles). Negative ratings, as analyzed by Wilcoxon rank-sum test refer to an improvement in the respective experience.

The Fig. 3 shows that overall daytime VMS did not significantly differ by therapy. Women perceived, however, that overall night sweats decreased more on progesterone than on placebo (*P* = 0.023). Progesterone also caused a greater perceived decrease in night sweat frequency (*P* = 0.015) and decreases in both night sweat (*P* < 0.001) and daytime VMS intensity (*P* < 0.014).

### Sleep, anxiety, menstrual flow, depression, and perimenopausal interference

**Figure 4:**
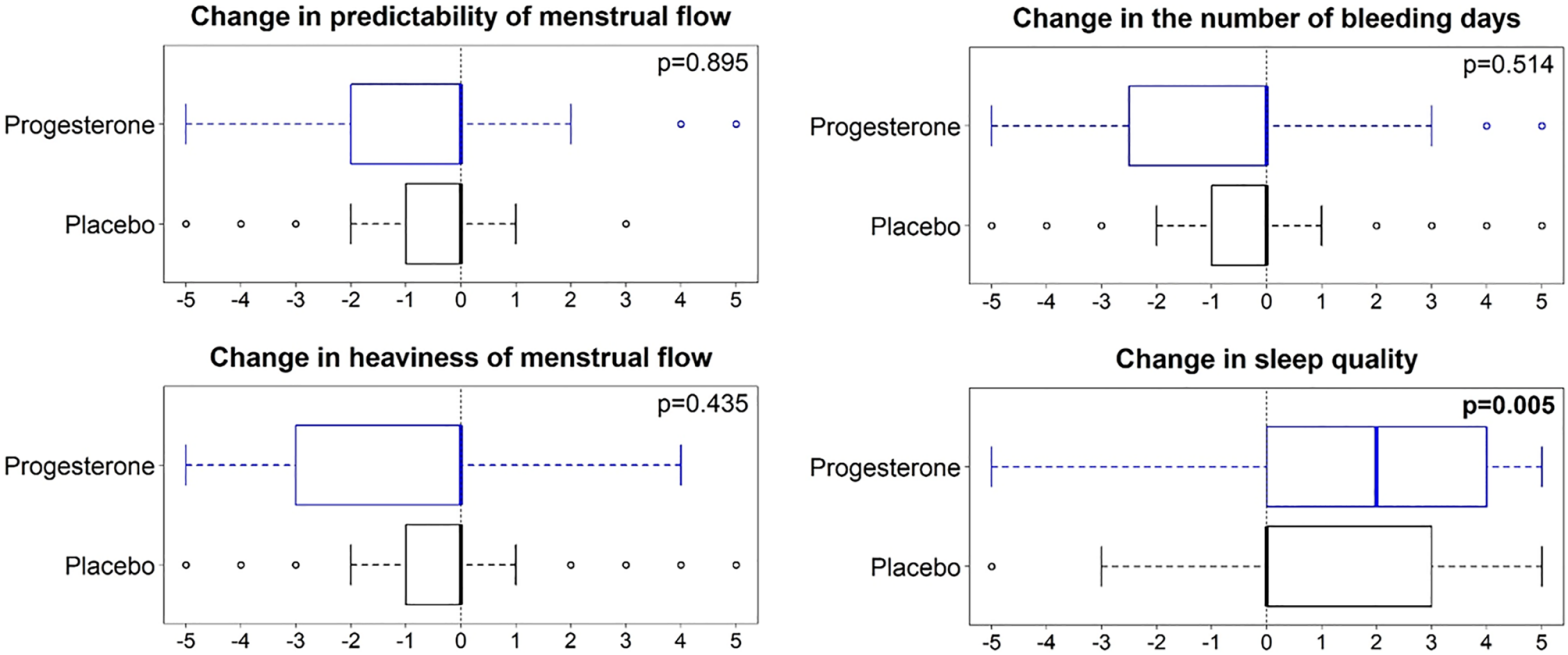
Perimenopausal women’s perceived changes in menstruation and sleep by progesterone (blue) or Placebo (black) 3-Month Randomized Therapy. The solid line is the median with the box including 50% (25th to 75th percentiles). Negative ratings, as analyzed by Wilcoxon rank-sum test, refer to decreased and positive relate to increased respective experiences.

Calendar records of “sleep problems” and “feelings of anxiety” were not different at the 3rd-m between women by randomization. However, women perceived that “sleep quality” was significantly improved on progesterone versus placebo (Fig. 4). More women on progesterone than placebo reported that their flow had stopped (28 vs. 14 percent, *P* = 0.024). However, perceived menstrual flow changes did not differ by therapy (Fig. 4).

The PHQ-9 (for depression) and Perimenopause Interference Questionnaire data did not differ by random therapy assignment at baseline (Table 1). Third-m PHQ-9 scores were not different between progesterone and placebo (Table 2). The mean 3rd-m Perimenopause Interference Questionnaire showed a significantly greater improvement (− 6.65 (95% CI − 12.11, − 1.18; *P* = 0.017) on progesterone than on placebo (Table 2).

Early and Late Perimenopause participants’ VMS and Perceived Change results are shown in Supplemental Table 1 and Supplemental Figure 2.

### Safety, adverse events and adherence

All adverse events were systematically collected monthly and blindly adjudicated for a potential relationship with progesterone therapy. No *serious* adverse events occurred during this trial (Table 3). There was one family physician-mandated discontinuation of progesterone treatment for new-onset atrial fibrillation; one progesterone-treated woman and four placebo-treated women discontinued due to ineffectiveness. One woman in each therapy group reported dizziness; this may have been due to progesterone. Although no statistically significant difference separated adverse events in the two groups (Table 3), collectively women on progesterone reported numerically more frequent mild-moderate “side effects” (n = 22; placebo n = 8). Medication adherence was excellent by capsule counts and Calendar records (91.4% on progesterone and 84.4% on placebo).

**Table 3:** Adverse events on oral micronized progesterone (300 mg at bedtime) or Placebo in perimenopausal women participating in a randomized double blind 12-Week trial for hot flushes and night sweats (n = 189).

	Adverse event	Progesterone N = 93	Control N = 96
1	Musculoskeletal, arthritis, connective tissue issues	6 (6.5%)	1 (1.1%)
2	Nausea, GI tract problems	8 (8.3%)	1 (1.1%)
3	Dizziness^	1 (1.0%)	1 (1.1%)
4	Edema, **atrial fibrillation, increased blood pressure, and headache	5 (5.2%)	3 (3.2%)
5	Worsening VMS	0	*1 (1.1%)
6	Post-traumatic stress	0	1 (1.1%)
7	Probable depression	1 (1.0%)	0
8	Facial acne	1 (1.0%)	0

*Discontinuation related to an adverse event on placebo.

**Family Physician-mandated discontinuation from progesterone experimental therapy.

^The only experience potentially related to progesterone therapy in blinded adjudication.

## Discussion

This four-month RCT of oral micronized progesterone versus placebo for hot flushes and/or night sweats (VMS) in almost 200 perimenopausal women found no significant 3rd-m improvement in the overall VMS Score or primary outcome. Data, however, could not *exclude* a minimal clinically important progesterone-related VMS benefit. An end-of-study questionnaire also documented that women randomized to progesterone perceived significant decreases in night sweats and in daytime VMS intensity. Progesterone significantly decreased perimenopausal women’s perception of physical and emotional interference with their daily activities^40^. Progesterone also significantly improved sleep quality, that, as well as VMS, is a treatment priority for symptomatic perimenopausal women^18^. Progesterone caused no perceived change in flow, was not associated with increased depression (by PHQ-9), and caused no serious adverse events.

VMS occur in both postmenopause and perimenopause. Although their pathophysiology remains to be fully understood, they appear to be triggered by acute downward swings in estrogen levels leading to increased central stress hormone releases and elevated central norepinephrine levels leading to a narrowed thermoneutral zone that causes heat dissipation symptoms^50^. Stressful social situations have also been shown experimentally to trigger VMS episodes^51^. Finally, a 13-year population-based prospective study, starting in 35-year old women with regular cycles, showed moderate-severe VMS had a 10-year mean duration that was longest when night sweats began in women with still-regular cycles^16^. Perimenopausal estradiol levels tend to be higher, are highly erratic and are not reliably suppressed by exogenous estrogen or progesterone levels^52^. Thus, evidence suggests perimenopausal VMS differ from, and *require a* unique therapy*, versus VMS that occur in postmenopause*.

This RCT was testing progesterone as a potentially effective VMS therapy in women in the menopause transition and perimenopause and showed it was effective for night sweats. Should perimenopausal women being treated for VMS with progesterone become postmenopausal, they could discontinue progesterone without experiencing a rebound in VMS^30^. They could also continue progesterone as needed for its VMS effectiveness given the RCT showing this in postmenopausal VMS^27^. When VMS are rare or absent for one year, a woman may stop progesterone to see whether they will return. There is also no concern with taking progesterone alone (without estrogen) since progesterone prevents endometrial cancer; it is estrogen-alone that poses a risk for endometrial cancer in a woman who has not had a hysterectomy.

Other trials of VMS have enrolled a few or some perimenopausal women^13–15^, and some had similar VMS eligibility criteria to this trial’s^13^, yet none has documented the high variability of perimenopausal women’s hot flushes and night sweats shown here. This is the first RCT also including perimenopausal *night sweats* that occurred for 100% of the women in Early Perimenopause. Despite conducting a pilot 4-month study, doing a pre-planned assessment of the SD of VMS half way through our initial recruitment, and excellent adherence, this trial lost 49 screened participants because their baseline month’s daily Calendar had insufficient VMS for randomization. This “improvement” between screening and the baseline may reflect perimenopausal VMS variability or the known benefits of research participation.

Funding played a role in study limitations. Funding was obtained for recruitment-extension for 1-year, however this timeline was still insufficient to achieve study completion of the 228 participants that were needed for adequate power. This study and the two previous perimenopause-only VMS RCT data were all underpowered^14,15^. The 32 women in an estradiol arm of a 1-year herbal RCT in perimenopausal/early-in-postmenopause women provided too few data to adequately assess its effectiveness or safety^13^. The current results are similar to a same-design RCT of progesterone for *postmenopausal* VMS in 133 healthy women that showed a statistically significant benefit and no serious adverse events^27^. Another study limitation is that we did not have serum hormonal characterization of participants at baseline.

The strengths of this RCT are that it meticulously followed Good Clinical Practice guidelines, and enrolled the largest number in a perimenopausal VMS trial participants to date. When local recruitment was slow, investigators transformed the design to offer and successfully accomplish remote participation with ethical accountability for experimental drugs and also achieved excellent participant retention (93%) and medication adherence. This trial, although designed a decade before the consensus on core outcomes for evaluating VMS^53^, assessed night sweats as well as hot flushes with daily frequency and intensity scores, and recorded all six core VMS trial outcomes except women’s satisfaction with therapy. Remote participation facilitated broad geographic inclusion and allowed the participation of busy women, thus extending generalizability. Finally, we clearly documented the greater variability of perimenopausal versus postmenopausal VMS making subsequent powered studies easier to plan.

Despite major efforts to learn the number of completed participants needed, to expand recruitment nationally, and to extend our RCT for one-year, we still were not able to randomize sufficient women. The results of this trial are not generalizable to all Canadian women since, despite a national catchment area, the majority of women were employed, higher educated, and White. Caution in interpretation of women’s night sweat improvements is needed since progesterone is RCT-documented to improve sleep^23,27^ and decrease sleep disturbances^24^. Sleep benefits cannot, however, explain the perceived improvement in intensity or severity of daytime VMS (*P* = 0.014). Also, given that VMS are experiential, results depended on the accuracy of both participants’ perceptions and their record-keeping.

Oral micronized progesterone therapy in perimenopausal women experiencing bothersome night sweats and/or hot flushes was inconclusive and did not show superiority over placebo in a three-month RCT. However, a minimum clinically important VMS progesterone benefit could not be excluded. Participants on progesterone perceived significantly greater decreases in overall night sweats and improved sleep quality versus those on placebo. Perimenopausal interference with daily activities also significantly decreased on daily progesterone therapy. Progesterone is biologically identical to the lower progesterone levels occurring in perimenopause^32^, is on most countries’ formularies, causes no increased thrombosis (as progestins do) and its primary pharmacological “adverse effect” is RCT-proven improvement in deep sleep^23,24,27^. Progesterone therapy may become especially applicable for perimenopausal women with frequent night sweats, sleep problems and difficulty coping^26^. A definitive, well-powered comparative perimenopausal VMS RCT of daily luteal phase-dose progesterone versus placebo or versus menopausal hormone therapy of estrogen with low dose progesterone/progestin is urgently needed.

### Supplementary Information

Supplementary Information 1.

Supplementary Information 2.

Supplementary Information 3.

## Data Availability

The datasets generated and/or analysed during the current study are not publicly available due to lack of informed consent but de-identified datasets are available from the corresponding author on reasonable request.
