# Author Correction: Oral micronized progesterone for perimenopausal night sweats and hot flushes a Phase III Canada-wide randomized placebo-controlled 4 month trial

**DOI:** 10.1038/s41598-024-68283-0

**Published:** 2024-07-27

**Authors:** Jerilynn C. Prior, Andrea Cameron, Michelle Fung, Christine L. Hitchcock, Patricia Janssen, Terry Lee, Joel Singer

**Affiliations:** 1 Centre for Menstrual Cycle and Ovulation Research, Endocrinology, University of British Columbia (UBC), 2775 Laurel Street, Suite 4111, Vancouver, BC Canada; 2 School of Population and Public Health, UBC, Vancouver, Canada; 3 Etobicoke General Hospital, William Osler Health System, Etobicoke, ON Canada; 4 British Columbia Women’s Health Research Institute, Vancouver, Canada; 5 Hitchcock Consulting, Oakville, ON Canada; 6 Centre for Health Evaluation and Outcome Sciences, Vancouver, BC Canada; 7 Endocrinology and Metabolism, Department of Medicine, UBC, Vancouver, BC Canada

Correction to: *Scientific Reports* 10.1038/s41598-023-35826-w, published online 05 June 2023

The original version of this Article contained errors in Table 2, where the values in the “All”, “Progesterone” and “Control” columns were incorrect for the Perimenopause Interference Questionnaire and Personal Health Questionnaire 9 (PHQ-9).

The correct and incorrect values appear below.

Incorrect:

**Table:** 

Variables	All (n = 189)	Progesterone (n = 93)	Control (n = 96)	RR (95% CI)* RD (95% CI)*	*P
Perimenopause Interference Questionnaire	5.1 (4.4)	4.8 (4.4)	5.4 (4.3)	− 6.65 (− 12.11, − 1.18)^^^	0.017
Personal Health Questionnaire 9 (PHQ-9)	25.3 (20.2)	22.6 (18.3)	28.2 (21.7)	− 0.40 (− 1.57, 0.77)^^^	0.501

Correct:

**Table:** 

Variables	All (n = 189)	Progesterone (n = 93)	Control (n = 96)	RR (95% CI)* RD (95% CI)*	*P
Perimenopause Interference Questionnaire	25.3 (20.2)	22.6 (18.3)	28.2 (21.7)	− 6.65 (− 12.11, − 1.18)^^^	0.017
Personal Health Questionnaire 9 (PHQ-9)	5.1 (4.4)	4.8 (4.4)	5.4 (4.3)	− 0.40 (− 1.57, 0.77)^	0.501

The original Article has been corrected.

