# Supplementary material for: Oral micronized progesterone for perimenopausal night sweats and hot flushes a Phase III Canada-wide randomized placebo-controlled 4 month trial
Source: Sci Rep. 2023 Jun 5;13:9082. doi: 10.1038/s41598-023-35826-w (PMC10241804; doi:10.1038/s41598-023-35826-w)
Supplement: Supplementary file 1 — Supplementary Information 1. [file 41598_2023_35826_MOESM1_ESM.pdf]

Supplemental Figure 1: Daily Perimenopause Hot Flush and Night Sweat Calendar

Daily Perimenopause Hot Flush Calendar

Study ID_______________ Month:______________Year: ______

Calendar Date	1	2	3	4	5	6	7	8	9	10	11	12	13	14	15	16	17	18	19	20	21	22	23	24	25	26	27	28	29	30	31
Tampons/pads/day																															

Record 0 = none, 1 = minimal, 2 = moderate, 3 = moderately intense, 4 = very intense

Hot flushes - day																															
# of flushes - day																															
Hot flushes - night																															
# of flushes - night																															
Sleep Problems																															
Feeling Anxious																															
Therapy (# pills)																															
Comments																															

Instructions:

1. **Calendar date**: Please record your experiences starting with whatever day of the month it is (not with the first day of your period).
2. **Tampons/pads/day**: Please record number of fully soaked regular-sized tampons/pads per day. (Example: 2 half-soaked tampons/pads equal one fully-soaked tampon/pad; one large or "maxi" pad = twice a regular-sized one. We know it's difficult—just do your best. . . .)
3. **Hot flushes – day**: This means you grade the ***hot flush intensity***. Please rate intensity of daytime hot flush using the scale above. (Example: 0 = you did not have any hot flushes, 1 = you barely noticed and it passed quickly, 2 = you had to react to it (take off a layer of clothing, fan yourself, drink cold water, etc.), 3 = you were sweating a little, 4 = you were sweating a lot ("a tropical storm!")
4. **# hot flushes – day**: Please record ***the number*** of daytime hot flushes you experienced.
5. **Hot flushes – night**: Please rate ***intensity*** of nighttime hot flushes (also called night sweats) using the scale above. Remember, a night sweat that woke you up is always rated at least a '2.' If you woke and went to the washroom, then realized you were sweating, that would also be '2'.
6. **# hot flushes – night**: Please record the ***number*** of nighttime hot flushes you experienced.
7. **Sleep problems**: Please rate the level of any difficulties with sleep on a 0-4 scale
8. **Feeling anxious**: Please rate your level of anxiety using a 0-4 scale.
9. **Therapy (# pills):** This refers to ***study medication***. During your baseline recording month, you will record '0' as you will not be taking study medication. During study treatment phase, you will be taking three round capsules so you will record '3' on each evening you took the pills.
10. **Comments**: Please use this section to record events in your life which you think may have affected your experiences (Example: stressful day at work, went away for the weekend, had the flu, etc.)

This is the truncated adaptation of the Daily Perimenopause Diary [4] used to acquire primary outcome data for the bulk of participants in this RCT.
