# Supplementary material for: Oral micronized progesterone for perimenopausal night sweats and hot flushes a Phase III Canada-wide randomized placebo-controlled 4 month trial
Source: Sci Rep. 2023 Jun 5;13:9082. doi: 10.1038/s41598-023-35826-w (PMC10241804; doi:10.1038/s41598-023-35826-w)
Supplement: Supplementary file 2 — Supplementary Information 2. [file 41598_2023_35826_MOESM2_ESM.pdf]

Supplemental Figure 2: Perceived Change in Day and Night Vasomotor Symptoms in All, Early and Late Perimenopausal Women by 12-Week Progesterone or Placebo Randomized Therapy.

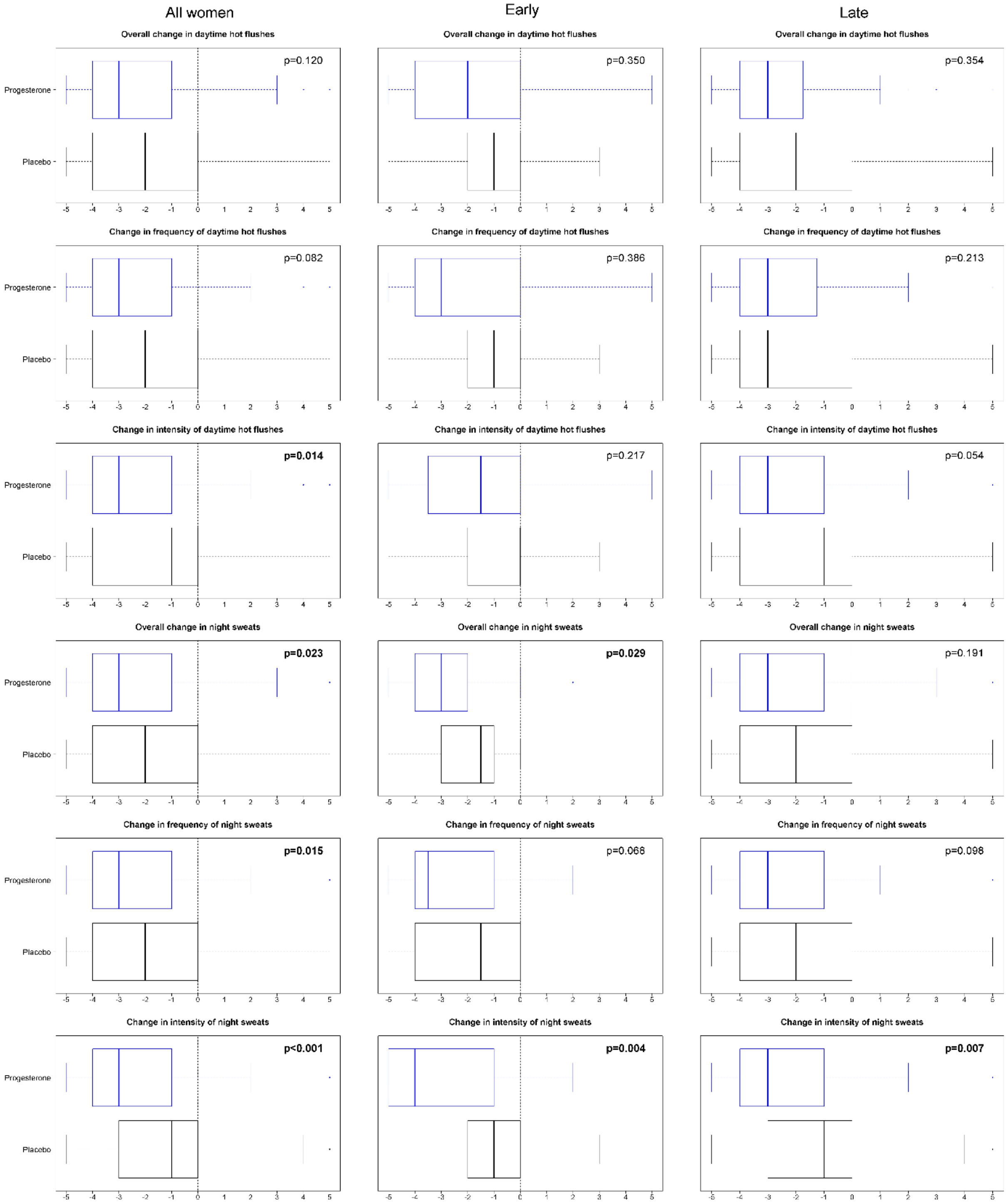

This box and whisker plot shows women's perceived changes in daytime hot flushes for all women in the Progesterone for Perimenopausal Night Sweats and Hot Flushes randomized controlled trial (left column) and separately for women in Early Perimenopause (regular or

irregular cycles) and in Late Perimenopause who had experienced a cycle length ≥60 days. Overall VMS change (based on VMS Score including number and intensity for both day and night vasomotor symptoms) is shown in the first row of plots. Negative ratings as analyzed by the Wilcoxon rank-sum test refer to an improvement in the respective experience.
