# Supplementary material for: Oral micronized progesterone for perimenopausal night sweats and hot flushes a Phase III Canada-wide randomized placebo-controlled 4 month trial
Source: Sci Rep. 2023 Jun 5;13:9082. doi: 10.1038/s41598-023-35826-w (PMC10241804; doi:10.1038/s41598-023-35826-w)
Supplement: Supplementary file 3 — Supplementary Information 3. [file 41598_2023_35826_MOESM3_ESM.docx]

**Supplemental Table 1. Vasomotor Symptom (VMS) Outcomes in Perimenopausal Women by “Early” and “Late”** (a ≥ 60 day cycle) **Randomized to Oral Micronized Progesterone or Placebo (Control) reported as VMS Score^#^, Frequency** (actual number/24-hour day**) and Intensity** (on a 0-4 scale in which ≥2 involves sweating) **in Intent-toTreat analysis.** Significant differences are in **bold**. Data are reported as mean and standard deviation (SD).

|  | **“Early” Perimenopause (n = 63)** | | | | **“Late” Perimenopause (n = 126)** | | | |
| --- | --- | --- | --- | --- | --- | --- | --- | --- |
| **Variable** | **All early**  **(n = 63)** | **Progesterone**  **(n = 29)** | **Control**  **(n = 34)** | ***P** | **All late**  **(n = 126)** | **Progesterone (n = 64)** | **Control**  **(n = 62)** | ***P** |
| ***VMS Score – run-in*** | 6.4 (5.9) | 6.3 (5.2) | 6.5 (6.4) | 0.929 | 15.1 (12.3) | 14.7 (11.3) | 15.5 (13.4) | 0.658 |
| ***VMS Score – 1^st^ 28 days*** | 5.6 (7.8) | 6.9 (10.2) | 4.6 (5.0) | 0.472 | 10.5 (11.8) | 9.2 (9.2) | 11.9 (14.1) | 0.678 |
| ***VMS Score – 2^nd^ 28 days*** | 3.8 (4.1) | 4.1 (4.7) | 3.6 (3.5) | 0.913 | 7.9 (10.8) | 5.8 (7.7) | 10.3 (13.0) | **0.015** |
| ***VMS Score – 3^rd^ 28 days*** | 4.2 (5.8) | 4.4 (7.4) | 3.9 (4.0) | 0.903 | 7.2 (10.5) | 5.9 (8.5) | 8.8 (12.3) | 0.150 |
| ***VMS frequency –***  ***run-in***  number per 24-hour day | 2.7 (2.1) | 2.7 (1.9) | 2.7 (2.2) | 0.915 | 6.0 (4.0) | 5.9 (3.7) | 6.1 (4.3) | 0.793 |
| ***VMS frequency –***  ***1^st^ 28 days***  number per 24-hour day | 2.3 (2.6) | 2.7 (3.3) | 2.0 (1.9) | 0.471 | 4.3 (3.9) | 3.8 (3.1) | 4.9 (4.7) | 0.516 |
| ***VMS frequency –***  ***2^nd^ 28 days***  number per 24-hour day | 1.8 (1.7) | 1.9 (1.9) | 1.7 (1.5) | 0.875 | 3.4 (3.6) | 2.7 (2.8) | 4.1 (4.2) | **0.038** |
| ***VMS frequency –***  ***3^rd^ 28 days***  number per 24-hour day | 1.8 (2.0) | 1.7 (2.2) | 1.9 (1.9) | 0.570 | 3.1 (3.6) | 2.6 (2.9) | 3.6 (4.3) | 0.207 |
| ***VMS intensity – run-in*** | 2.0 (0.7) | 2.0 (0.8) | 2.0 (0.6) | 0.870 | 2.4 (0.7) | 2.4 (0.7) | 2.4 (0.6) | 0.687 |
| ***VMS intensity – 1^st^ 28 days*** | 1.6 (1.0) | 1.7 (1.1) | 1.5 (0.9) | 0.442 | 1.9 (0.9) | 1.9 (1.0) | 1.9 (0.9) | 0.841 |
| ***VMS intensity – 2^nd^ 28 days*** | 1.4 (0.9) | 1.4 (1.0) | 1.4 (0.8) | 0.952 | 1.6 (1.0) | 1.4 (1.0) | 1.8 (1.0) | 0.061 |
| ***VMS intensity – 3^rd^ 28 days*** | 1.3 (1.0) | 1.3 (1.1) | 1.4 (0.9) | 0.717 | 1.5 (1.1) | 1.4 (1.0) | 1.6 (1.1) | 0.419 |

^#^VMS Score is the daytime number X intensity plus the nighttime number X intensity

*Based on negative binomial regression adjusted for log of the average daily run-in VMS Score (when applicable) and with the inclusion of an interaction term between perimenopause phase and treatment group. Please refer Statistical Considerations.
